# Complete Genome Sequence of *Rhodococcus* sp. Strain WMMA185, a Marine Sponge-Associated Bacterium

**DOI:** 10.1128/genomeA.01406-16

**Published:** 2016-12-15

**Authors:** Navid Adnani, Doug R. Braun, Bradon R. McDonald, Marc G. Chevrette, Cameron R. Currie, Tim S. Bugni

**Affiliations:** a Pharmaceutical Sciences Division, University of Wisconsin–Madison, Madison, Wisconsin, USA; b Department of Bacteriology, University of Wisconsin–Madison, Madison, Wisconsin, USA; c Laboratory of Genetics, University of Wisconsin–Madison, Madison, Wisconsin, USA

## Abstract

The *Rhodococcus* strain WMMA185 was isolated from the marine sponge *Chondrilla nucula* as part of ongoing drug discovery efforts. Analysis of the 4.44-Mb genome provides information regarding interspecies interactions as pertains to regulation of secondary metabolism and natural product biosynthetic potentials.

## GENOME ANNOUNCEMENT

Drug resistance is now commonplace for microbial human pathogens and approaches to discovering new antibiotics, indeed, completely new antibacterial chemotypes, continue to be developed to counter this problem (1). Accordingly, the number of natural products (as possible drug leads) available for screening, their producers and their production conditions must necessarily increase. In support of this tenet, the production of natural products by one producing organism in the presence of another organism has garnered tremendous interest (2–6). Such co-culturing of microorganisms has proven extremely effective for coaxing microbes into making natural products that would not otherwise be produced. This approach to new natural product generation underscores the importance of having genomic information available for co-cultured organisms (6). Importantly, the structural diversity of natural products enabled by co-culturing stems, in large part, from the diversity of co-cultured organisms.

The genus *Rhodococcus* is a Gram-positive bacterium within the subgroup actinobacteria whose members are commonly associated with bioremediation and biocatalytic processes (7–9); steroids, nitriles, lignins, and organosulfur agents are but a few compound classes degraded by rhodococci (10). Additionally, although rare, select *Rhodococcus* spp. are human pathogens; pathogenicity has been associated with similarities to *Mycobacterium* spp. (11, 12).

Genome analyses of the few reported *Rhodococcus* spp. highlight tremendous biosynthetic potential despite a scarcity of isolated secondary metabolites (13). Recently, cocultures of *Rhodococcus* spp. with other actinobacteria, including *Streptomyces* spp. (14, 15) and *Micromonosporaceae* (2) have been shown to produce otherwise undetectable secondary metabolites. In particular, marine invertebrate-associated *Rhodococcus* sp. WMMA185 induced biosynthesis in other marine actinobacteria via interspecies interactions; the precise nature of these interactions awaits further investigation. Using genomic data from WMMA185, mechanisms of biosynthetic regulation and interspecies communication may be deciphered in an effort to access unexploited (or cryptic) biosynthetic potentials from actinobacteria or, for that matter, WMMA185 itself.

*Rhodococcus* sp. strain WMMA185 was isolated in 2011 from a marine sponge *Chondrilla nucula* collected off the coast of the Florida Keys. WMMA185 was isolated from a plate prepared using R2A medium supplemented with 50% artificial seawater (ASW).

The complete genome of *Rhodococcus* sp. WMMA185 was sequenced at the Duke Center for Genomic and Computational Biology (GCB) using PacBio RS II (Pacific Biosciences) technology. Reads were assembled using the HGAP assembler (16) into a single contig. Open reading frames were predicted by Prodigal (17) and annotated using HMMer models for the TIGRfam (18), KEGG (19, 20), and PFAM (21, 22) databases. The genome was found to be 4.44 Mb in length and has 64.08% G+C, and 90.39% coding density. The organism’s secondary metabolic content/potential was assessed using anti-SMASH (23, 24), PRISM (25), and custom pipelines. Among other cluster types, a total of two type I polyketide (PKS), eight nonribosomal peptide (NRPS), and two terpene biosynthetic gene clusters were identified within the WMMA185 genome.

### Accession number(s).

The complete genome sequence of *Rhodococcus* sp. strain WMMA185 has been deposited at the DDBJ/EMBL/GenBank under the sequence GenBank accession no. CP017014.
